# Supplementary material for: Lipid Profile, Eating Habit, and Physical Activity in Children with Down Syndrome: A Prospective Study
Source: Diseases. 2024 Mar 29;12(4):68. doi: 10.3390/diseases12040068 (PMC11049486; doi:10.3390/diseases12040068)

Figure S1. Variation of LDL at baseline visit and 2-year follow-up visit.

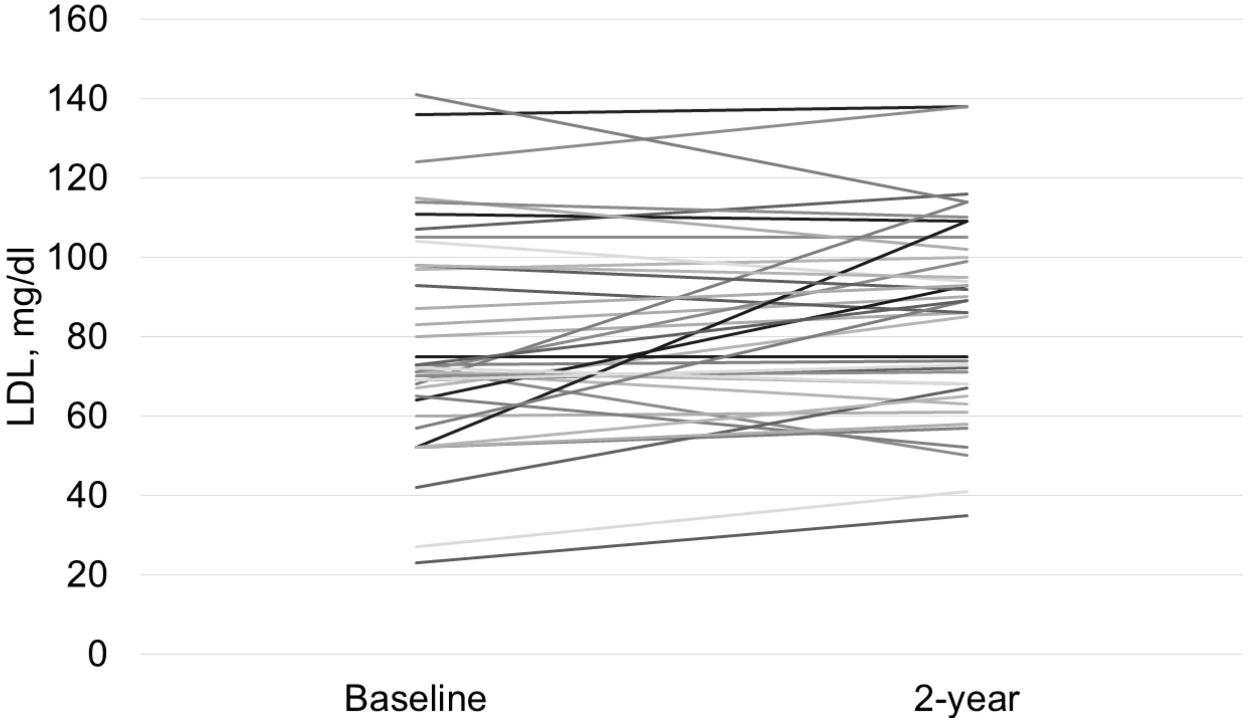

Figure S2. Variation of HDL at baseline visit and 2-year follow-up visit.

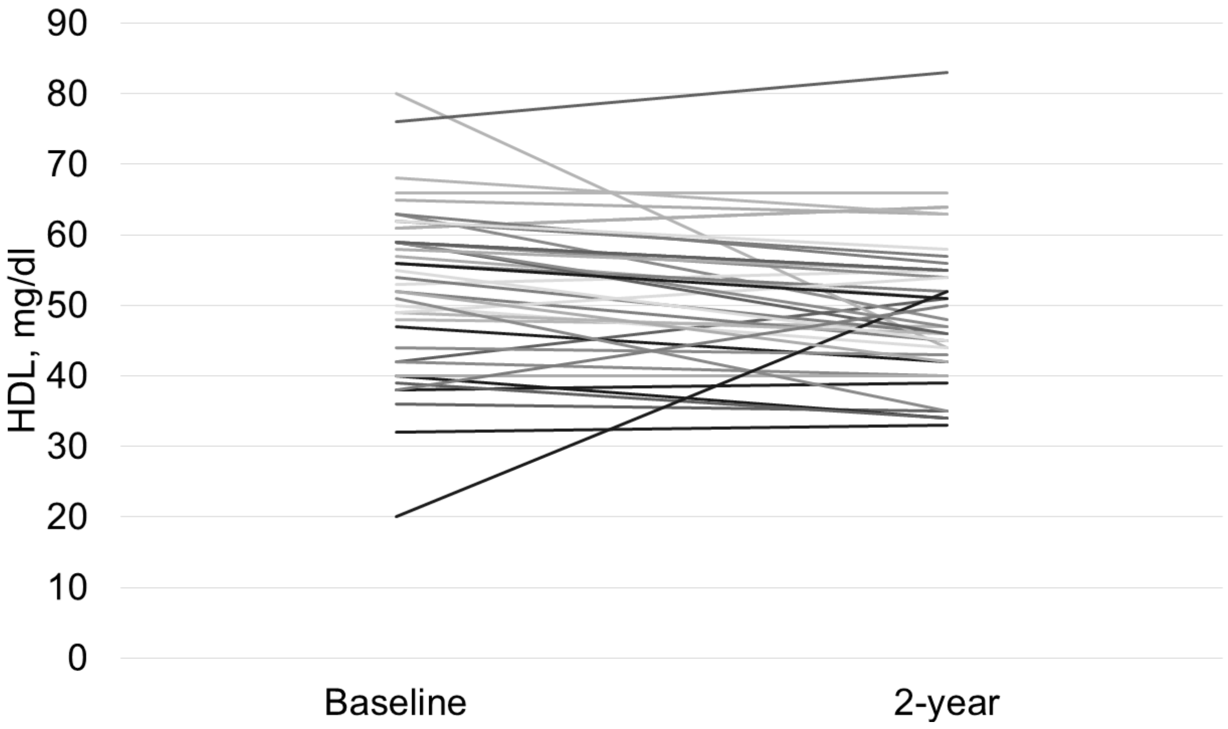

Figure S3. Variation of TGC at baseline visit and 2-year follow-up visit.

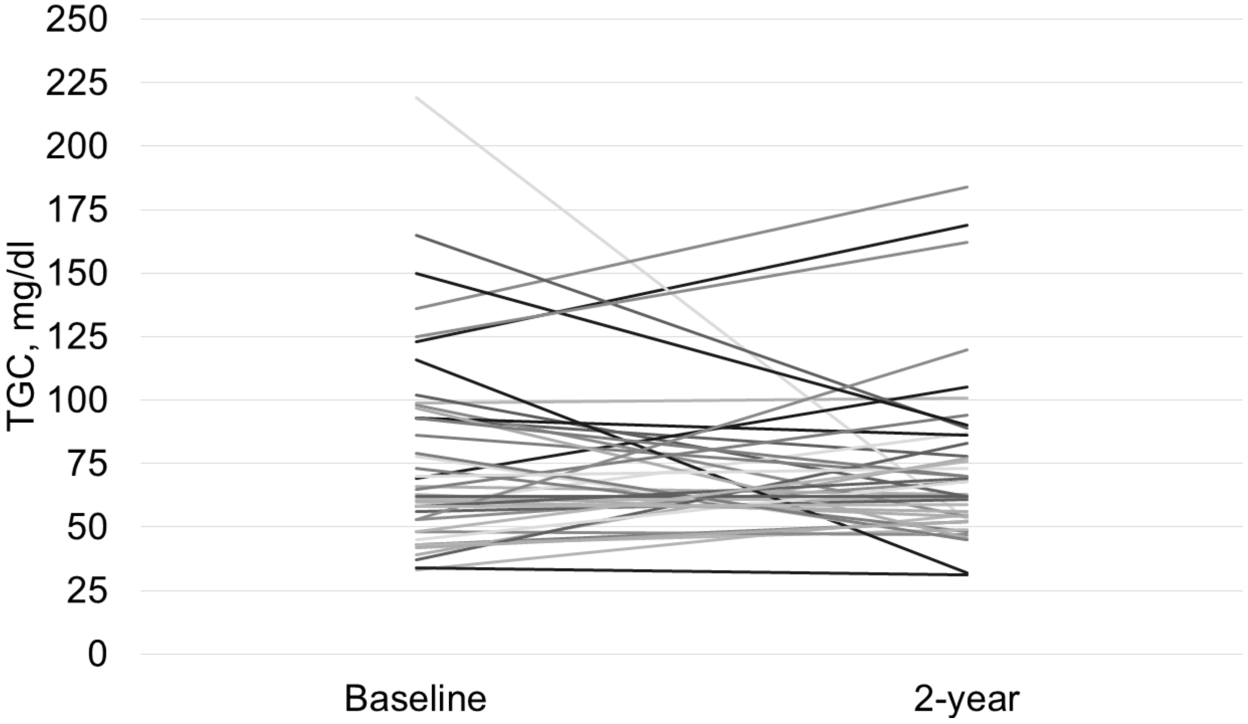

Supplement: Supplementary file 1 [file diseases-12-00068-s001.zip › diseases-2899275-supplementary.pdf]
